# Variants in autophagy‐related genes and clinical characteristics in melanoma: a population‐based study

**DOI:** 10.1002/cam4.929

**Published:** 2016-10-17

**Authors:** Kirsten A. M. White, Li Luo, Todd A. Thompson, Salina Torres, Chien‐An Andy Hu, Nancy E. Thomas, Jenna Lilyquist, Hoda Anton‐Culver, Stephen B. Gruber, Lynn From, Klaus J. Busam, Irene Orlow, Peter A. Kanetsky, Loraine D. Marrett, Richard P. Gallagher, Lidia Sacchetto, Stefano Rosso, Terence Dwyer, Anne E. Cust, Colin B. Begg, Marianne Berwick, The GEM Study Group, Urvi Mujumdar, Pampa Roy, Bruce Armstrong, Anne Kricker, Melisa Litchfield, Paul Tucker, Alison Venn, Nicola Stephens, Teresa Switzer, Loraine Marrett, Elizabeth Theis, Noori Chowdhury, Louise Vanasse, Roberto Zanetti, Carlotta Sacerdote, Nancy Leighton, Joanne Jeter, Judith Klotz, Homer Wilcox, Helen Weiss, Dianne Mattingly, Jon Player, Timothy Rebbeck, Amy Walker, Saarene Panossian, Julia Lee Taylor, Sasha Madronich

**Affiliations:** ^1^ Department of MedicineDivision of EpidemiologyUniversity of New MexicoAlbuquerqueNew Mexico; ^2^ Department of Pharmaceutical SciencesCollege of PharmacyUniversity of New MexicoAlbuquerqueNew Mexico; ^3^ Center for HPV PreventionDepartment of Pathology University of New MexicoAlbuquerqueNew Mexico; ^4^ Department of Biochemistry and Molecular BiologyUniversity of New MexicoAlbuquerqueNew Mexico; ^5^ Department of DermatologyUniversity of North CarolinaChapel HillNorth Carolina; ^6^ Lineberger Comprehensive Cancer CenterUniversity of North CarolinaChapel HillNorth Carolina; ^7^ Department of EpidemiologySchool of MedicineUniversity of CaliforniaIrvineCalifornia; ^8^ Department of MedicineKeck School of MedicineUniversity of Southern CaliforniaLos AngelesCalifornia; ^9^ Cancer Care OntarioTorontoOntarioCanada; ^10^ Department of PathologyMemorial Sloan Kettering Cancer CenterNew YorkNew York; ^11^ Department of Cancer EpidemiologyH. Lee Moffitt Cancer Center & Research InstituteTampaFlorida; ^12^ British Columbia Cancer AgencyVancouverBritish ColumbiaCanada; ^13^ Piedmont Cancer RegistryCentre for Epidemiology and Prevention in Oncology in PiedmontTurinItaly; ^14^ George Institute for Global HealthUniversity of OxfordUK; ^15^ University of SydneySydneyNew South WalesAustralia

**Keywords:** *ATG16L1*, autophagy, melanoma, polymorphism, SNP

## Abstract

Autophagy has been linked with melanoma risk and survival, but no polymorphisms in autophagy‐related (*ATG*) genes have been investigated in relation to melanoma progression. We examined five single‐nucleotide polymorphisms (SNPs) in three *ATG* genes (*ATG5*;*ATG10*; and *ATG16L*) with known or suspected impact on autophagic flux in an international population‐based case–control study of melanoma. DNA from 911 melanoma patients was genotyped. An association was identified between (GG) (rs2241880) and earlier stage at diagnosis (OR 0.47; 95% Confidence Intervals (CI) = 0.27–0.81, *P *=* *0.02) and a decrease in Breslow thickness (*P *=* *0.03). The *ATG16L* heterozygous genotype (AG) (rs2241880) was associated with younger age at diagnosis (*P *=* *0.02). Two SNPs in *ATG5* were found to be associated with increased stage (rs2245214 CG, OR 1.47; 95% CI = 1.11–1.94, *P *=* *0.03; rs510432 CC, OR 1.84; 95% CI = 1.12–3.02, *P *=* *0.05). Finally, we identified inverse associations between *ATG5* (GG rs2245214) and melanomas on the scalp or neck (OR 0.20, 95% CI = 0.05–0.86, *P *=* *0.03); *ATG10* (CC) (rs1864182) and brisk tumor infiltrating lymphocytes (TILs) (OR 0.42; 95% CI = 0.21–0.88, *P *=* *0.02), and *ATG5* (CC) (rs510432) with nonbrisk TILs (OR 0.55; 95% CI = 0.34–0.87, *P *=* *0.01). Our data suggest that *ATG* SNPs might be differentially associated with specific host and tumor characteristics including age at diagnosis, TILs, and stage. These associations may be critical to understanding the role of autophagy in cancer, and further investigation will help characterize the contribution of these variants to melanoma progression.

## Introduction

Autophagy is one mechanism of tumorigenesis that is under intensive investigation. This catabolic process assists the removal of unnecessary or dysfunctional cellular components, including damaged proteins and organelles through lysosomal degradation 1. Autophagy is tightly regulated, plays a role in a wide variety of normal physiological processes including energy metabolism, stress responses, growth regulation, and aging 2, 3, and can be induced in response to nutrient deprivation 4. Macroautophagy (hereafter referred to as autophagy) digests dysfunctional macromolecules and damaged organelles. Accumulating evidence indicates that autophagy is involved in cancer development and progression 3, 5, and the notion that melanomas are addicted to autophagy 5, 6, 7, 8, 9 has important implications for cancer development as well as management of treatment options for this difficult disease 10. The American Cancer Society estimates that in 2016, 76,380 new melanomas will be diagnosed in the United States and 10,130 people will die from their disease 11. The long‐term prognosis for melanoma patients has not improved at the same rate as other cancers 12.

There are several clinical trials currently ongoing at the National Institutes of Health to examine targeting inhibition of the autophagic pathway in multiple cancer types including melanoma 13. However, the extent to which the rate of autophagic flux impacts melanoma development and progression remains to be elucidated.

Single‐nucleotide polymorphisms (SNPs) have been found to be associated with risk and/or prognosis in numerous cancers including breast, thyroid, prostate, colorectal, and gastric cancer 14, 15, 16, 17. However, to our knowledge, there are no studies examining the relationship between *ATG* SNPs and stage or histopathological markers in melanoma. We hypothesized that variants in *ATG* genes may affect gene expression and ultimately influence the rate of autophagic flux and impact melanoma progression. To test this hypothesis, we analyzed germline DNA samples for variants (i.e., SNPs) in three *ATG* genes in a population‐based cohort of melanoma patients. The SNPs investigated were chosen for having a functional impact on disease risk and/or progression and have been identified as significantly associated in the current literature with disease outcomes and ≥10% minor allele frequency in Caucasians 15, 18, 19, 20, 21. In particular, the SNP in *ATG16L* (rs2241880) has been reported to create a caspase cleavage site in ATG16L, resulting in an unstable protein and decreased autophagy. Importantly, this autophagy SNP has been shown as causative for Crohn's disease 22.

## Materials and Methods

A total of 3,578 individuals with melanoma from nine study sites including eight population‐based cancer registries in the United States (New Jersey, North Carolina, and California), Australia (New South Wales and Tasmania), Canada (Ontario and British Columbia), and Italy (Turin), and one hospital center in Michigan were enrolled in the Genes, Environment and Melanoma (GEM), a large international population‐based study. GEM recruitment procedures and data collection have been previously described 23. The Institutional Review Boards of all participating institutions approved the protocol and written informed consent was obtained from each participant.

### Participant selection

From a total of 1206 individuals with multiple primary melanoma and 2372 with single primary melanoma, 911 participants who had extracted DNA available for genotyping and for whom tumor tissue was currently available (for purposes of future functional studies) were selected (Table 1) 24. DNA was isolated from buccal cells as previously described 23, 25. A Nanodrop 2000 spectrophotometer (ThermoFisher Scientific, Grand Island, NY) was used for quantification of DNA.

**Table 1:** Clinicopathologic characteristics among 911 melanoma cases

Characteristic		No.(%)
Median age at diagnosis	60 years	
Median Breslow thickness	0.8 mm	
Gender		
Male		534 (59)
Female		377 (41)
Breslow thickness (mm)		
0.01–1.00		547 (60)
1.01–2.00		212 (23)
2.01–4.00		108 (12)
>4.00		44 (5)
Status		
Single primary		603 (66)
Multiple primary		308 (34)
Anatomic site		
Trunk/pelvis		394 (43)
Scalp/neck		56 (6)
Face/ears/other		116 (13)
Upper extremities		172 (19)
Lower extremities		173 (19)
Histological subtype		
Superficial Spreading Melanoma		610 (67)
Nodular Melanoma		92 (10)
Lentigo Maligna Melanoma		116 (13)
Other		93 (10)
Ulceration		
Absent		794 (92)
Present		73 (8)
Missing		44 (0)
Mitosis		
Absent		454 (52)
Present		415 (48)
Missing		42 (0)
AJCC stage		
T1a		397 (46)
T1b		124 (14)
T2a		183 (21)
T2b		16 (2)
T3a		73 (8)
T3b		32 (4)
T4a		21 (2)
T4b		21 (2)
AJCC stage (T1a/T1b/T2a vs. T2b+)		
T1a/T1b/T2a		704 (81)
T2b+		163 (19)
TIL grade		
Absent		194 (22)
Non‐Brisk		563 (65)
Brisk		111 (13)
Missing		43 (0)
Growth phase		
Absent		255 (29)
Present		614 (71)
Missing		42 (0)
Death from melanoma*		76 (8)
Alive or death from other causes		835 (92)

AJCC, American Joint Committee on Cancer; TIL, Tumor infiltrating lymphocytes; *Death from melanoma recorded during 7.5 years of follow‐up.

### Clinical Stage

Histopathology slides were reviewed as previously described 26. Mitoses were defined as present or absent; and tumor infiltrating lymphocytes (TIL) grade was scored as absent, nonbrisk, or brisk using a previously defined grading system 26. We used T classification, which describes the state of the primary tumor in the American Joint Committee on Cancer (AJCC) TNM (tumor, regional nodes, and distant metastasis) melanoma staging system to determine tumor stage based on Breslow thickness, mitotic index, and ulceration.

### Selection of SNPs and genotyping

**Figure 1:**
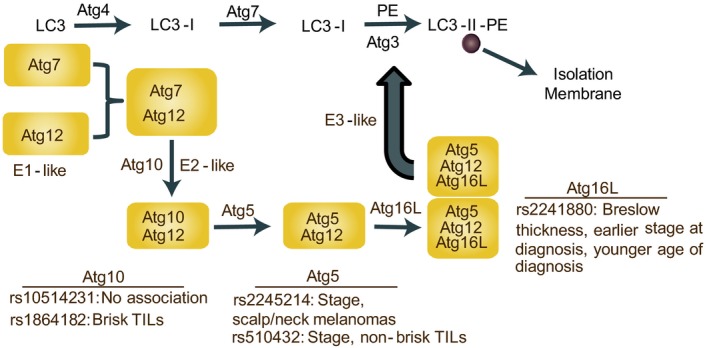
Overview of the Autophagy related (ATG) conjugation pathway including significant associations with SNPs investigated in this study, including those in the *ATG5*,* ATG10* and *ATG16L* genes.

Five SNPs in three critical *ATG* genes (*ATG5* rs2245214 C >G rs510432 T >C; *ATG10* rs1864182 A >C, rs1051423 T >C; *ATG16L* rs2241880 A>G) were selected from functional SNPs in the literature or that were associated with cancer or disease outcomes 15, 18, 19, 21, 27 (see Fig. 1). Five Taqman Real‐Time PCR Genotyping Assays (ThermoFisher Scientific, Grand Island, NY) were used to identify SNPs in *ATG* genes performed with a 7900HT Fast Real‐Time PCR System (ThermoFisher Scientific, Grand Island, NY) following manufacture recommendations. The ratio of fluorescence in amplification during the logarithmic phase was quantified to identify specific alleles in genes of interest using a commercially available Taqman primer assay on a 7900HT Applied Biosystems qPCR machine. The genotyping call rates ranged from 96% to 99%, and biological replicates were generated for 10% of the samples with 100% concordance.

### Data analysis

To assess genotyping quality, we calculated the genotype call rates and tested the departure from Hardy‐Weinberg Equilibrium for each SNP. SNPs were modeled as a three‐level nominal variable. Linear regression analyses were performed to assess the association between SNPs and log‐transformed Breslow thickness. We used the log‐transformed Breslow thickness to correct for the nonnormal distribution and back‐transformed model coefficients so that results represent increases in Breslow thickness per 1 mm. To evaluate the association between *ATG* SNPs and histopathological features, we conducted binary logistic regression analyses for mitosis (presence or absence), ulceration (presence or absence), early versus late stage (early‐ T1a/T1b/T2a vs. late‐stage T2b and higher), ordinal logistic regression for continuous stage (T1a though T4b), and polytomous logistic regression for histology and tumor subtype. Odds Ratios (ORs) and 95% Confidence Intervals (CI) estimated from logistic regression models are presented. We conducted multivariable modeling to account for covariates and Wald tests were used to assess the significance of the association. Statistical tests were two sided with *P *<* *0.05 considered statistically significant. Using a genotypic model to simultaneously compare heterozygous genotype to wildtype, and homozygous minor genotype to wildtype, we report a global *P*‐value representing the overall significance of the two comparisons for analysis.

## Results

Genotype frequencies are presented in Table 2, and genotypes did not deviate from Hardy‐Weinberg proportions (data not shown). The distribution of SNP genotypes at the five *ATG* genes was similar in males and females (data not shown). After adjustments for age, gender, status (SPM or MPM) and study center, three *ATG* SNPs—rs2241880, rs510432, rs2245214—were inversely associated with several melanoma prognostic indicators (Tables 3, 4 and Table S2; Fig. 1). In separate analyses of each SNP with Breslow thickness, the *ATG16L* rs2241880 GG genotype was associated with a decrease in Breslow thickness (*P *=* *0.02), earlier stage at diagnosis (OR 0.47; 95% CI = 0.27–0.81, *P *=* *0.02). Additionally, rs2241880 heterozygosity (AG) was associated with a younger age of diagnosis (*P *=* *0.02).

**Table 2:** Genotypic frequencies of *ATG* gene polymorphisms tested in melanoma cases

*ATG* SNP	Genotype	Number of patients (%)
*ATG5*		
rs510432	CC	190 (0.22)
	CT	425 (0.48)
	TT	266 (0.30)
Missing		30
*ATG5*		
rs2245214	CC	331 (0.38)
	CG	427 (0.49)
	GG	110 (0.13)
Missing		43
*ATG16L*		
rs2241880	AA	198 (0.23)
	AG	418 (0.490
	GG	245 (0.29)
Missing		50
*ATG10*		
rs10514231	CC	116 (0.13)
	CT	403 (0.47)
	TT	345 (0.40)
Missing		47
*ATG10*		
rs1864182	AA	238 (0.28)
	AC	424 (0.49)
	CC	200 (0.23)
Missing		49

**Table 3:** Associated clinicopathologic characteristics by genotype status among melanoma cases

Age at diagnosis, y					
*ATG* gene SNP	Genotype^a^	*n*	Coeff (95% CI)	*P*‐value*	Global *P*‐value*
rs10514231	TT	864	1.00		
	CT		0.22 (−1.95–2.39)	0.84	0.71
	CC		1.33 (−1.84–4.50)	0.41	
rs1864182	AA	862	1.00		
	AC		0.72 (−1.67–3.11)	0.55	0.70
	CC		−0.26 (−3.09–2.57)	0.86	
rs2241880	AA	861	1.00		
	AG		−3.25 (−5.60–0.91)	**0.01**	**0.02**
	GG		−2.04 (−4.83–0.74)	0.15	
rs2245214	CC	868	1.00		
	CG		−0.59 (−2.77–1.58)	0.59	0.75
	GG		−1.17 (−4.43–2.09)	0.48	
rs510432	TT	881	1.00		
	CT		0.43 (−1.86–2.73)	0.71	0.75
	CC		−0.55 (−3.33–2.23)	0.70	

Breslow thickness (Continuous; Exponentiated)				
*ATG* gene SNP	Genotype^b^	Coeff (95% CI)	*P*‐value*	Global *P*‐value*
rs10514231	TT	1.00		0.18
	CT	1.09 (0.98–1.22)	0.09	
	CC	0.99 (0.84–1.16)	0.91	
rs1864182	AA	1.00		0.72
	AC	1.05 (0.92–1.19)	0.46	
	CC	1.05 (0.9–0.82)	0.50	
rs2241880	AA	1.00		**0.03**
	AG	1.04 (0.92–0.85)	0.55	
	GG	0.87 (0.97–0.99)	0.06	
rs22445214	CC	1.00		0.28
	CG	1.09 (0.98–1.22)	0.11	
	GG	1.06 (0.9–1.26)	0.49	
rs510432	TT	1.00		0.30
	CT	1.05 (0.94–1.19)	0.37	
	CC	1.12 (0.97–1.3)	0.12	

CI, confidence interval; Coeff, coefficient; *P < .05 were considered significant; Bolded results indicate significant associations.

a Genotypic model adjusted for gender, study center. and status.

b Genotypic model adjusted for age (continuous), gender, study center, and status.

**Table 4:** Relationship Between *ATG* Genotype and AJCC Stage in Melanoma

Melanoma Stage(≥ Stage T2b vs. Stage T1a/T1b/T2a)				
*ATG* gene SNP	Genotype	≥ Stage T2b versus Stage T1a/T1b/T2a	*P*‐value*	Global *P*‐value*
rs10514231	TT	1.00		
	CT	1.18 (0.80–1.76)	0.41	0.46
	CC	0.84 (0.46–1.54)	0.57	
rs1864182	AA	1.00		
	AC	1.22 (0.79–1.89)	0.38	0.52
	CC	0.98 (0.57–1.66)	0.93	
rs2241880	AA	1.00		
	AG	0.88 (0.59–1.33)	0.55	**0.02**
	GG	0.47 (0.27–0.81)	**0.01**	
rs2245214	CC	1.00		
	CG	1.46 (0.98–2.17)	0.06	0.14
	GG	1.05 (0.57–1.92)	0.88	
rs510432	TT	1.00		
	CT	1.26 (0.81–1.95)	0.30	**0.05**
	CC	1.84 (1.12–3.02)	**0.02**	

Melanoma Stage(≥ Stage T2b vs. Stage T1a/T1b/T2a)				
*ATG* gene SNP	Genotype	OR (95% CI)(Continuous Stage)	*P*‐value*	Global *P*‐value*
rs10514231	TT	1 [Reference]		
	CT	1.18 (0.90–1.56)	0.23	0.32
	CC	0.92 (0.61–1.38)	0.69	
rs1864182	AA	1 [Reference]		
	AC	1.07 (0.79–1.46)	0.65	0.90
	CC	1.06 (0.74–1.53)	0.74	
rs2241880	AA	1 [Reference]		
	AG	1.01 (0.75–1.37)	0.93	0.14
	AA	0.74 (0.52–1.06)	0.10	** **
rs2245214	CC	1 [Reference]		
	CG	1.47 (1.11–1.94)	**0.01**	**0.03**
	GG	1.23 (0.82–1.85)	0.32	
rs510432	TT	1 [Reference]		
	CT	1.30 (0.97–1.75)	0.08	0.14
	CC	1.37 (0.96–1.95)	0.09	** **

Genotypic model adjusted for age (continuous), gender, study center, and status. Abbreviations: AJCC, American Joint Committee on Cancer; *P values < .05 were considered significant; Bolded results indicate significant associations.

*ATG5* rs2245214 (AA) and *ATG5* rs510432 (CC) were positively associated with later stage (OR 1.47; 95% CI = 1.11–1.94, *P *=* *0.03; OR 1.84; 95% CI = 1.12–3.02, *P *=* *0.05) (Table 4). The homozygous variant (CC) of rs510432 also had a borderline association with later stage.

SNP rs1864182 (CC) and rs510432 (CC) were inversely associated with brisk TILs (OR 0.42; 95% CI = 0.21–0.88, *P *=* *0.02; OR 0.55; 95% CI = 0.34–0.87, *P *=* *0.01, respectively) as well as the presence of nonbrisk TILs (Table S2). Finally, rs2245214 (GG) was inversely associated with scalp/neck melanomas (OR 0.20; 95% CI = 0.05–0.86, *P *=* *0.03), although there was not a global association of this SNP with anatomic site of melanoma (Table S2). No associations between the five autophagy SNPs and mitosis, ulceration, or histological subtype (Table S2) were identified. We also did not identify an association between any of the five SNPs and melanoma survival.

## Discussion

Despite clear associations between autophagy and cancer etiology 28, 29, 30, the role of germline SNPs in melanoma stage at diagnosis has remained unexplored. Autophagy in cancer is context dependent, acting as both a tumor suppressor and tumor promotor depending on the stage of development of the tumor 31. While a recent meta‐analysis of GWAS studies did not observe an association between *ATG* gene SNPs and melanoma susceptibility 32, we know of no other study specifically addressing the associations between common genetic variants in *ATG* genes and melanoma survival.

The SNPs investigated in our study are located in genes that are critical to the early stage of the autophagy pathway (Fig. 1) and necessary for the formation of the autophagosome 1. As shown in Figure 1, *ATG10* is essential for *ATG12* conjugation to *ATG5* and ultimately to *ATG16L*.

Previously, variants in *ATG* genes have been associated with risk and/or prognosis in other cancers 15, 18, 19 and autoimmune conditions 14, 22, 33. In this study, we examined one SNP (rs2241880) in *ATG16L*, which increased risk for thyroid cancer and was associated with poor disease prognosis. A nonsynonymous polymorphism in *ATG16L*, rs2241880 (T300A), has been extensively studied in Crohn's disease 34. This *ATG16L* SNP (GG) creates a caspase 3 and caspase 7 cleavage site and reduces the stability of the protein resulting in decreased autophagy; clinically, presence of this variant is associated with increased risk of ileal Crohn's disease in adults and decreased survival 34. While this SNP is associated with increased susceptibility, it is also associated with childhood (early) onset of Crohn's disease 35. As illustrated in Figure 1, *ATG16L* is essential for the formation of the autophagosome. Through its noncovalent interaction with *ATG12–ATG5,* it facilitates the conjugation of other critical ATG proteins. Two SNPs in *ATG5* (rs2241880 and rs2245214) have been associated with a nearly twofold susceptibility to nonmedullary thyroid cancers 19 and rs2241880 is associated with disease severity 18 as well as two‐fold risk of developing colorectal cancer 16.

ATG5 is part of an ubiquitin‐like conjugation pathway which links ATG5 with ATG16L (ATG5‐ATG16L). Specifically, ATG5 membrane binding is activated through its conjugation with ATG16L. Membrane binding by the ATG12–ATG5–ATG16 exerts an E3 enzyme‐like function and this binding is critical for the correct formation of the autophagosome (Fig. 1). Importantly, both rs1864182 and rs1051423, located in ATG10, have been reported to be associated with a *decreased* risk of breast cancer 15.

In this study, three SNPs were associated with melanoma prognostic indicators: Breslow thickness, stage at diagnosis, and TILs. In *ATG16L* rs 2241880 (GG) was associated with decreased Breslow thickness (*P *=* *0.03) and earlier stage at diagnosis (OR 0.47; 95% CI = 0.27–0.81, *P *=* *0.02). *ATG16L* rs 2241880 (AG) was also associated with younger age at diagnosis (*P *=* *0.02). This SNP is also associated in the literature with decreased autophagy and may mediate melanoma progression through the accumulation of protein aggregates and damaged organelles in patients 36, 37. There is some evidence that decreased autophagy may inhibit melanoma tumorgeneis 5, 6. Furthermore, this *ATG16L* SNP has been associated with increased IL‐1*β* production in primary cells 34. Metastatic melanoma cells spontaneously secrete active IL‐1*β* 38 and the association between melanoma and this *ATG* variant warrants further investigation.

In *ATG5,* two SNPs, rs510432 (CC) (OR 1.84; 95% CI = 1.12–3.02, *P *=* *0.05) and rs2245214 (CG) (OR 1.47; 95% CI = 1.11–1.94, *P *=* *0.03), were associated with increased stage. SNP rs510432 had a borderline association with nonbrisk TILs (OR 0.55; 95% CI = 0.34–0.87, *P *=* *0.01), although not significant at the global *P*‐value. Interestingly, rs510432 is located in the 5′ untranslated region (UTR) upstream of its first exon in the promotor region. In addition, this SNP (rs510432) (CC) is associated with asthma (*P *=* *0.003)^27^ conferring significantly increased promotor activity. As we also identified a positive association with increased stage and rs510432 (CC) in our population, further studies exploring the functional role of this SNP in the rate of autophagy and melanoma progression may elucidate whether the promoter of *ATG5* has increased activity in these participants, leading to more advanced stage.

In addition, *ATG5* has functions independent of autophagy, including critical roles in apoptosis, mitotic catastrophe, and regulation of the *β*‐Catenin signaling pathway 39, 40, 41. As *ATG5* is often downregulated in primary melanomas 42, the association of two SNPs in this critical *ATG* gene with increased melanoma stage is significant as they have the potential to become new markers of melanoma risk, progression, and/or therapeutic targets.

No significant associations were identified between the five SNPs and ulceration, mitosis, or histological subtype. However, while they were not significant at the level of the global *P*‐value, rs1864182 (CC) and rs510432 (CC) were inversely associated with brisk TILs (OR 0.42; 95% CI = 0.21–0.88, *P *=* *0.02; OR 0.55; 95% CI = 0.34–0.87, *P *=* *0.01), as well as the presence of nonbrisk TILs. The association of TILs with autophagy variants is important because higher TIL grade in primary melanomas is associated with improved melanoma‐specific survival 43. In addition, autophagy's role in modulation of the immune system could have important implications for immunotherapy, although the effect of this intersection and the role of *ATG* gene variants on TIL grade require further investigation.

Finally, while not significant at the global *P*‐value, an inverse association between the homozygous genotype (GG) of rs2245214 and scalp/neck melanomas was also identified (OR 0.20; 95% CI = 0.05–0.86, *P *=* *0.03). As it has been previously documented that individuals with scalp/neck melanomas have poorer outcomes than patients with melanomas on other sites 44, this inverse relationship warrants further studies to determine if there is a functional significance for *ATG5* and this anatomic site.

Autophagy has an established role in cancer; however, the relationship between genetic variants in autophagy genes and melanoma risk and/or progression remains under explored. In this study, we assessed the impact of variants in critical *ATG* genes that are necessary for autophagic flux in relationship with melanoma prognostic indicators and survival. Drugs targeting the autophagy pathway are currently being investigated as effective therapy for many cancers including melanoma. SNPs that alter autophagic rates may impact the effectiveness of current treatment strategies and thus have clinical significance 7, 30, 45, 46, 47, 48. In silico analysis of results from multiple studies, and/or coordination of large studies, will be required to assess the reproducibility of these *ATG* gene interactions in melanoma.

This study is limited by the knowledge that alteration of autophagy might not be due to variants in *ATG* genes, but possibly due to other signaling pathways that regulate autophagy or posttranslational modifications. In addition, there are probably other functional genetic variants not included in this study, as there are approximately 38 *ATG* genes specifically required for autophagy in the yeast model *Saccharomyces cerevisiae* 49. We did not find a direct association between any of the five *ATG* gene SNPs and survival, although this may be due to insufficient sample size. Our analyses did not control for multiple comparisons, such as false discovery rate. These limitations will have to be addressed in future studies, including screening for SNPs in other relevant genes using alternative technologies, such as deep sequencing, to identify variants of interest as well as measuring changes in ATG protein levels due to the impact of these SNPs. However, our study should be considered exploratory based on the fact that it was designed with an a priori hypothesis that genetic variants in the autophagy pathway would modify risk of melanoma. It should be pointed out that our findings confirmed and support other reports addressing the impact of these variants in cancer risk as presented in the literature and highlight the need for additional studies evaluating the functional significance of these SNPs.

In conclusion, we have identified three *ATG* gene SNPs as genetic factors impacting melanoma progression, which, in melanoma patients, may result in changes in ATG protein levels and alter autophagy regulation, impacting melanomagenesis. These findings emphasize the significance of the autophagy pathway in melanoma. As the role of autophagy in melanoma is complex and context dependent, the reported associations may provide important insight into how SNPs in critical autophagy genes impact melanoma progression.

## Conflict of Interest

None declared.

## Supporting information


**Table S1.** Power Analysis between *ATG* Genotype and AJCC Stage in Melanoma.
**Table S2.** Nonsignificant clinicopathologic characteristics by genotype status among melanoma cases.

## Appendix 1

The study was conducted by the GEM Study Group: Marianne Berwick (PI, University of New Mexico), Memorial Sloan Kettering Cancer Center, New York, NY: Colin Begg (Co‐PI), Irene Orlow (Co‐Investigator), Urvi Mujumdar (Project Coordinator), Klaus Busam (Dermatopathologist), Pampa Roy (Laboratory Technician). Study Centers: The University of Sydney and The Cancer Council New South Wales, Sydney (Australia): Bruce Armstrong (PI), Anne Kricker (co‐PI), Melisa Litchfield (Study Coordinator). Menzies Research Institute, University of Tasmania, Hobart (Australia): Terence Dwyer (PI, currently at the Murdoch Childrens Research Institute, Melbourne, Victoria), Paul Tucker (Dermatopathologist), Alison Venn (co‐Investigator), Nicola Stephens (Study Coordinator). British Columbia Cancer Agency, Vancouver (Canada): Richard Gallagher (PI), Teresa Switzer (Coordinator). Cancer Care Ontario, Toronto (Canada): Loraine Marrett (PI), Elizabeth Theis (Co‐Investigator), Lynn From (Dermatopathologist), Noori Chowdhury (Coordinator), Louise Vanasse (Coordinator). Centro per la Prevenzione Oncologia Torino, Piemonte (Italy): Stefano Rosso (PI), Roberto Zanetti (co‐PI), Carlotta Sacerdote (Coordinator). University of California, Irvine, CA: Hoda Anton‐Culver (PI), Nancy Leighton (Coordinator). University of Michigan, Ann Arbor, MI: Stephen Gruber (PI), Joanne Jeter (Coordinator). New Jersey Department of Health and Senior Services, Trenton, NJ: Judith Klotz (PI), Homer Wilcox (Co‐PI), Helen Weiss (Coordinator). University of North Carolina, Chapel Hill, NC: Robert Millikan (PI), Nancy Thomas (Co‐Investigator), Dianne Mattingly (Coordinator), Jon Player (Laboratory Technician). University of Pennsylvania, Philadelphia, PA: Timothy Rebbeck (PI), Peter Kanetsky (Co‐Investigator), Amy Walker (Laboratory Manager), Saarene Panossian (Laboratory Technician). Consultants: Julia Lee Taylor and Sasha Madronich, National Centre for Atmospheric Research, Boulder, CO.
