# Supplementary material for: Variants in autophagy‐related genes and clinical characteristics in melanoma: a population‐based study
Source: Cancer Med. 2016 Oct 17;5(11):3336–45. doi: 10.1002/cam4.929 (PMC5119988; doi:10.1002/cam4.929)
Supplement: Supplementary file 1 — Table S1. Power Analysis between ATG Genotype and AJCC Stage in Melanoma. Table S2. Nonsignificant clinicopathologic characteristics by genotype status among melanoma cases. [file CAM4-5-3336-s001.docx]

| **Supplemental Table 1.** Power Analysis between *ATG* Genotype and AJCC Stage in Melanoma |
| --- |

The association between *ATG* gene SNPs and melanoma stage (T1a-T2a vs. T2b-higher). For a sample size of N=911, with the significance level of 0.05 and 80% power, the minimum detectable per allele odds ratio are described in the following table:

| *ATG* gene SNP | MAF | Minimum Detectable Odds Ratio |  |
| --- | --- | --- | --- |
| **rs10514231** | 0.367 | 1.41 |  |
| **rs1864182** | 0.478 | 1.40 |  |
| **rs2241880** | 0.473 | 1.40 |  |
| **rs2245214** | 0.373 | 1.41 |  |
| **rs510432** | 0.457 | 1.40 |  |
|  |  |  |  |
| **Supplemental Table 2.** Non-significant clinicopathologic characteristics by genotype status among melanoma cases | | | |

| **TIL Grade** |  |  |  |  |  |
| --- | --- | --- | --- | --- | --- |

| *ATG* gene SNP | Genotype | | TIL Grade | | Coeff (95%CI) | | | p-value* | | | Global p-value* | | | |  | |  |
| --- | --- | --- | --- | --- | --- | --- | --- | --- | --- | --- | --- | --- | --- | --- | --- | --- | --- |
| **rs10514231** | | TT | Absent | 1 [Reference] | | | | |  | | | 0.88 | | |  |  |  |
|  | | CT | Non-Brisk | 0.98 (0.68-1.41) | | | | | 0.91 | | |  |  |  |  |  |  |
|  | | CT | Brisk | 0.92 (0.54-1.59) | | | | | 0.78 | | |  |  |  |  |  |  |
|  | | CC | Non-Brisk | 1.15 (0.67-1.98) | | | | | 0.62 | | |  |  |  |  |  |  |
|  | | CC | Brisk | 0.80 (0.34-1.86) | | | | | 0.60 | | |  |  |  |  |  |  |
| **rs1864182** | | CC | Absent | 1 [Reference] | | | | |  | | | 0.14 | | |  |  |  |
|  | | AC | Non-Brisk | 1.12 (0.74-1.68) | | | | | 0.60 | | |  |  |  |  |  |  |
|  | | AC | Brisk | 0.87 (0.49-1.58) | | | | | 0.66 | | |  |  |  |  |  |  |
|  | | AA | Non-Brisk | 0.81 (0.51-1.29) | | | | | 0.37 | | |  |  |  |  |  |  |
|  | | AA | Brisk | 0.42 (0.21-0.88) | | | | | **0.02** | | |  |  |  |  |  |  |
| **rs2241880** | | GG | Absent | 1 [Reference] | | | | |  | | | 0.42 | | |  |  |  |
|  | | AG | Non-Brisk | 0.91 (0.61-1.35) | | | | | 0.63 | | |  |  |  |  |  |  |
|  | | AG | Brisk | 1.02 (0.55-1.90) | | | | | 0.96 | | |  |  |  |  |  |  |
|  | | AA | Non-Brisk | 0.89 (0.55-1.43) | | | | | 0.62 | | |  |  |  |  |  |  |
|  | | AA | Brisk | 1.53 (0.76-3.08) | | | | | 0.23 | | |  |  |  |  |  |  |
| **rs2245214** | | TT | Absent | 1 [Reference] | | | | |  | | | 0.97 | | |  |  |  |
|  | | CG | Non-Brisk | 0.93 (0.65-1.34) | | | | | 0.70 | | |  |  |  |  |  |  |
|  | | CG | Brisk | 0.86 (0.50-1.49) | | | | | 0.59 | | |  |  |  |  |  |  |
|  | | CC | Non-Brisk | 0.91 (0.53-1.57) | | | | | 0.74 | | |  |  |  |  |  |  |
|  | | CC | Brisk | 1.01 (0.46-2.22) | | | | | 0.98 | | |  |  |  |  |  |  |
| **rs510432** | | TT | Absent | 1 [Reference] | | | | |  | | | 0.12 | | |  |  |  |
|  | | CT | Non-Brisk | 0.74 (0.49-1.11) | | | | | 0.15 | | |  |  |  |  |  |  |
|  | | CT | Brisk | 0.99 (0.54-1.79) | | | | | 0.96 | | |  |  |  |  |  |  |
|  | | CC | Non-Brisk | 0.55 (0.34-0.87) | | | | | **0.01** | | |  |  |  |  |  |  |
|  | | CC | Brisk | 0.72 (0.36-1.45) | | | | | 0.36 | | |  |  |  |  |  |  |
| **Ulceration** | |  |  |  | | | | |  | | |  | | |  |  |  |
| *ATG* gene SNP | | Genotype | Ulceration (Absent is referent) | | | | p-value* | | Global p-value* | | | | |  |  | |  |
| **rs10514231** | | CC | 1 [Reference] | | |  | | | 0.82 | | | |  | |  |  |  |
|  | | CT | 1.04 (0.60-1.81) | | | 0.90 | | |  |  |  |  |  | |  |  |  |
|  | | TT | 0.80 (0.35-1.86) | | | 0.61 | | |  |  |  |  |  | |  |  |  |
| **rs1864182** | | AA | 1 [Reference] | | |  | | | 0.61 | | | |  | |  |  |  |
|  | | AC | 1.23 (0.66-2.29) | | | 0.52 | | |  |  |  |  |  | |  |  |  |
|  | | CC | 0.90 (0.41-1.95) | | | 0.78 | | |  |  |  |  |  | |  |  |  |
| **rs2241880** | | AA | 1 [Reference] | | |  | | | 0.41 | | | |  | |  |  |  |
|  | | AG | 0.94 (0.53-1.68) | | | 0.84 | | |  |  |  |  |  | |  |  |  |
|  | | GG | 0.61 (0.28-1.31) | | | 0.20 | | |  |  |  |  |  | |  |  |  |
| **rs2245214** | | CC | 1 [Reference] | | |  | | | 0.29 | | | |  | |  |  |  |
|  | | CG | 1.33 (0.77-2.28) | | | 0.31 | | |  |  |  |  |  | |  |  |  |
|  | | GG | 0.70 (0.27-1.78) | | | 0.45 | | |  |  |  |  |  | |  |  |  |
| **rs510432** | | CC | 1 [Reference] | | |  | | | 0.36 | | | |  | |  |  |  |
|  | | CT | 1.27 (0.68-2.37) | | | 0.45 | | |  |  |  |  |  | |  |  |  |
|  | | TT | 1.68 (0.83-3.43) | | | 0.15 | | |  |  |  |  |  | |  |  |  |
| **Mitosis** | |  |  |  | | | | |  | | | |  | |  |  |  |
| *ATG* gene SNP | | Genotype | Mitosis (Absent is referent) | | | p-value* | | | Global p-value* | | | | |  | |  | |
| **rs10514231** | | CC | 1 [Reference] | | |  | | | | 0.84 | | | |  | |  |  |
|  | | CT | 1.09 (0.81-1.48) | | | 0.56 | | | |  |  |  |  |  | |  |  |
|  | | TT | 1.02 (0.65-1.58) | | | 0.95 | | | |  |  |  |  |  | |  |  |
| **rs1864182** | | AA | 1 [Reference] | | |  | | | | 0.50 | | | |  | |  |  |
|  | | AC | 0.95 (0.68-1.33) | | | 0.77 | | | |  |  |  |  |  | |  |  |
|  | | CC | 1.17 (0.79-1.74) | | | 0.43 | | | |  |  |  |  |  | |  |  |
| **rs2241880** | | AA | 1 [Reference] | | |  | | | | 0.49 | | | |  | |  |  |
|  | | AG | 1.06 (0.76-1.48) | | | 0.73 | | | |  |  |  |  |  | |  |  |
|  | | GG | 0.86 (0.58-1.27) | | | 0.44 | | | |  |  |  |  |  | |  |  |
| **rs2245214** | | CC | 1 [Reference] | | |  | | | | 0.17 | | | |  | |  |  |
|  | | CG | 1.33 (0.98-1.79) | | | 0.07 | | | |  |  |  |  |  | |  |  |
|  | | GG | 1.30 (0.83-2.03) | | | 0.25 | | | |  |  |  |  |  | |  |  |
| **rs510432** | | CC | 1 [Reference] | | |  | | | | 0.34 | | | |  | |  |  |
|  | | CT | 1.18 (0.86-1.63) | | | 0.31 | | | |  |  |  |  |  | |  |  |
|  | | TT | 1.33 (0.90-1.96) | | | 0.15 | | | |  |  |  |  |  | |  |  |
| **Anatomic site of lesion** | | |  |  | | | | |  | | | |  | |  |  |  |
| *ATG* gene SNP | | Genotype | Site of Lesion | | | Coeff (95% CI) | | | p-value* | | | | | Global p-value* | |  |  |
|  | |  |  |  | | | | |  | | | |  | |  |  |  |
| **rs10514231** | | TT | Trunk/pelvis | 1 [Reference] | | | | |  | | | | 0.73 | |  |  |  |
|  | | CT | Scalp/neck | 0.74 (0.39-1.39) | | | | | 0.34 | | | |  |  |  |  |  |
|  | | CT | Face/ears/other | 1.06 (0.65-1.74) | | | | | 0.80 | | | |  |  |  |  |  |
|  | | CT | Upper extremities | 0.8 (0.53-1.2) | | | | | 0.28 | | | |  |  |  |  |  |
|  | | CT | Lower extremities | 0.97 (0.63-1.47) | | | | | 0.87 | | | |  |  |  |  |  |
|  | | CC | Scalp/neck | 0.46 (0.15-1.4) | | | | | 0.17 | | | |  |  |  |  |  |
|  | | CC | Face/ears/other | 1.36 (0.71-2.62) | | | | | 0.35 | | | |  |  |  |  |  |
|  | | CC | Upper extremities | 0.76 (0.41-1.42) | | | | | 0.39 | | | |  |  |  |  |  |
|  | | CC | Lower extremities | 0.97 (0.52-1.82) | | | | | 0.92 | | | |  |  |  |  |  |
| **rs1864182** | | CC | Trunk/pelvis | 1 [Reference] | | | | |  | | | | 0.22 | |  |  |  |
|  | | AC | Scalp/neck | 1.56 (0.77-3.14) | | | | | 0.22 | | | |  |  |  |  |  |
|  | | AC | Face/ears/other | 1 (0.58-1.71) | | | | | 0.99 | | | |  |  |  |  |  |
|  | | AC | Upper extremities | 0.79 (0.5-1.24) | | | | | 0.31 | | | |  |  |  |  |  |
|  | | AC | Lower extremities | 0.68 (0.25-1.81) | | | | | 0.44 | | | |  |  |  |  |  |
|  | | AA | Scalp/neck | 0.83 (0.52-1.33) | | | | | 0.44 | | | |  |  |  |  |  |
|  | | AA | Face/ears/ other | 1.33 (0.72-2.47) | | | | | 0.37 | | | |  |  |  |  |  |
|  | | AA | Upper extremities | 0.94 (0.55-1.62) | | | | | 0.83 | | | |  |  |  |  |  |
|  | | AA | Lower extremities | 1.26 (0.73-2.17) | | | | | 0.41 | | | |  |  |  |  |  |
| **rs2241880** | | GG | Trunk/pelvis | 1 [Reference] | | | | |  | | | | 0.70 | |  |  |  |
|  | | AG | Scalp/neck | 1.68 (0.83-3.4) | | | | | 0.15 | | | |  |  |  |  |  |
|  | | AG | Face/ears/other | 0.9 (0.53-1.52) | | | | | 0.68 | | | |  |  |  |  |  |
|  | | AG | Upper extremities | 0.96 (0.62-1.5) | | | | | 0.87 | | | |  |  |  |  |  |
|  | | AG | Lower extremities | 0.99 (0.62-1.59) | | | | | 0.97 | | | |  |  |  |  |  |
|  | | AA | Scalp/neck | 0.88 (0.34-2.27) | | | | | 0.79 | | | |  |  |  |  |  |
|  | | AA | Face/ears/ other | 1.18 (0.65-2.16) | | | | | 0.59 | | | |  |  |  |  |  |
|  | | AA | Upper extremities | 0.96 (0.57-1.63) | | | | | 0.88 | | | |  |  |  |  |  |
|  | | AA | Lower extremities | 1.09 (0.63-1.89) | | | | | 0.75 | | | |  |  |  |  |  |
| **rs2245214** | | TT | Trunk/pelvis | 1 [Reference] | | | | |  | | | | 0.51 | |  |  |  |
|  | | CG | Scalp/neck | 0.73 (0.4-1.33) | | | | | 0.31 | | | |  |  |  |  |  |
|  | | CG | Face/ears/other | 0.9 (0.56-1.45) | | | | | 0.66 | | | |  |  |  |  |  |
|  | | CG | Upper extremities | 0.73 (0.49-1.1) | | | | | 0.14 | | | |  |  |  |  |  |
|  | | CG | Lower extremities | 0.84 (0.55-1.28) | | | | | 0.41 | | | |  |  |  |  |  |
|  | | CC | Scalp/neck | 0.2 (0.05-0.86) | | | | | **0.03** | | | |  |  |  |  |  |
|  | | CC | Face/ears/other | 0.88 (0.44-1.77) | | | | | 0.72 | | | |  |  |  |  |  |
|  | | CC | Upper extremities | 0.68 (0.37-1.26) | | | | | 0.22 | | | |  |  |  |  |  |
|  | | CC | Lower extremities | 0.72 (0.38-1.37) | | | | | 0.32 | | | |  |  |  |  |  |
| **rs510432** | | TT | Trunk/pelvis | 1 [Reference] | | | | |  | | | | 0.20 | |  |  |  |
|  | | CT | Scalp/neck | 0.65 (0.35-1.23) | | | | | 0.18 | | | |  |  |  |  |  |
|  | | CT | Face/ears/other | 0.67 (0.41-1.11) | | | | | 0.12 | | | |  |  |  |  |  |
|  | | CT | Upper extremities | 1.27 (0.82-1.98) | | | | | 0.28 | | | |  |  |  |  |  |
|  | | CT | Lower extremities | 1.23 (0.78-1.95) | | | | | 0.38 | | | |  |  |  |  |  |
|  | | CC | Scalp/neck | 0.45 (0.19-1.07) | | | | | 0.07 | | | |  |  |  |  |  |
|  | | CC | Face/ears/other | 0.68 (0.37-1.24) | | | | | 0.21 | | | |  |  |  |  |  |
|  | | CC | Upper extremities | 0.88 (0.51-1.52) | | | | | 0.65 | | | |  |  |  |  |  |
|  | | CC | Lower extremities | 0.98 (0.56-1.71) | | | | | 0.94 | | | |  |  |  |  |  |
| **Histological sub-type** | | |  |  | | | | |  | | | |  | |  |  |  |
| *ATG* gene SNP | Genotype | | Site of Lesion | | Coeff (95%CI) | | | p-value* | | | Global p-value* | | | |  | |  |
| **rs10514231** | | TT | SSM | 1 [Reference] | | | | |  | | | | 0.36 | |  |  |  |
|  | | CT | NM | 1.21 (0.70-2.07) | | | | | 0.50 | | | |  |  |  |  |  |
|  | | CT | LMM | 1.08 (0.68-1.73) | | | | | 0.74 | | | |  |  |  |  |  |
|  | | CT | Other | 0.95 (0.59-1.54) | | | | | 0.83 | | | |  |  |  |  |  |
|  | | CC | NM | 1.78 (0.90-3.52) | | | | | 0.10 | | | |  |  |  |  |  |
|  | | CC | LMM | 1.10 (0.57-2.13) | | | | | 0.77 | | | |  |  |  |  |  |
|  | | CC | Other | 0.46 (0.19-1.14) | | | | | 0.09 | | | |  |  |  |  |  |
| **rs1864182** | | CC | SSM | 1 [Reference] | | | | |  | | | | 0.46 | |  |  |  |
|  | | AC | NM | 1.58 (0.85-2.94) | | | | | 0.15 | | | |  |  |  |  |  |
|  | | AC | LMM | 1.52 (0.90-2.57) | | | | | 0.12 | | | |  |  |  |  |  |
|  | | AC | Other | 1.00 (0.59-1.71) | | | | | 0.99 | | | |  |  |  |  |  |
|  | | AA | NM | 1.63 (0.81-3.27) | | | | | 0.17 | | | |  |  |  |  |  |
|  | | AA | LMM | 1.12 (0.59-2.11) | | | | | 0.73 | | | |  |  |  |  |  |
|  | | AA | Other | 0.80 (0.41-1.56) | | | | | 0.52 | | | |  |  |  |  |  |
| **rs2241880** | | GG | SSM | 1 [Reference] | | | | |  | | | | 0.54 | |  |  |  |
|  | | AG | NM | 1.63 (0.92-2.88) | | | | | 0.09 | | | |  |  |  |  |  |
|  | | AG | LMM | 1.31 (0.78-2.19) | | | | | 0.30 | | | |  |  |  |  |  |
|  | | AG | Other | 0.89 (0.53-1.51) | | | | | 0.66 | | | |  |  |  |  |  |
|  | | AA | NM | 1.18 (0.60-2.36) | | | | | 0.63 | | | |  |  |  |  |  |
|  | | AA | LMM | 1.18 (0.65-2.15) | | | | | 0.59 | | | |  |  |  |  |  |
|  | | AA | Other | 0.74 (0.39-1.42) | | | | | 0.36 | | | |  |  |  |  |  |
| **rs2245214** | | TT | SSM | 1 [Reference] | | | | |  | | | | 0.26 | |  |  |  |
|  | | CG | NM | 1.59 (0.95-2.67) | | | | | 0.08 | | | |  |  |  |  |  |
|  | | CG | LMM | 0.88 (0.56-1.39) | | | | | 0.59 | | | |  |  |  |  |  |
|  | | CG | Other | 1.41 (0.84-2.37) | | | | | 0.20 | | | |  |  |  |  |  |
|  | | CC | NM | 0.76 (0.31-1.85) | | | | | 0.54 | | | |  |  |  |  |  |
|  | | CC | LMM | 0.80 (0.40-1.60) | | | | | 0.53 | | | |  |  |  |  |  |
|  | | CC | Other | 1.40 (0.68-2.90) | | | | | 0.36 | | | |  |  |  |  |  |
| **rs510432** | | TT | SSM | 1 [Reference] | | | | |  | | | | 0.96 | |  |  |  |
|  | | CT | NM | 1.03 (0.60-1.78) | | | | | 0.91 | | | |  |  |  |  |  |
|  | | CT | LMM | 0.88 (0.54-1.43) | | | | | 0.60 | | | |  |  |  |  |  |
|  | | CT | Other | 0.89 (0.53-1.49) | | | | | 0.65 | | | |  |  |  |  |  |
|  | | CC | NM | 1.33 (0.71-2.49) | | | | | 0.38 | | | |  |  |  |  |  |
|  | | CC | LMM | 0.98 (0.54-1.78) | | | | | 0.94 | | | |  |  |  |  |  |
|  | | CC | Other | 0.93 (0.49-1.76) | | | | | 0.83 | | | |  |  |  |  |  |

Genotypic model adjusted for age (continuous) sex, study center and status. Abbreviations: CI, confidence interval; Coeff, coefficient
